# Hemoglobin A1c Levels and Aortic Arterial Stiffness: The Cardiometabolic Risk in Chinese (CRC) Study

**DOI:** 10.1371/journal.pone.0038485

**Published:** 2012-08-03

**Authors:** Jun Liang, Na Zhou, Fei Teng, Caiyan Zou, Ying Xue, Manqing Yang, Huaidong Song, Lu Qi

**Affiliations:** 1 Department of Endocrinology, the Central Hospital of Xuzhou, Affiliated Hospital of Southeast University, Xuzhou Clinical School of Xuzhou Medical College, Xuzhou, Jiangsu, China; 2 Department of Nutrition, Harvard School of Public Health, Boston, Massachusetts, United States of America; 3 Channing Laboratory, Department of Medicine, Brigham and Women's Hospital and Harvard Medical School, Boston, Massachusetts, United States of America; 4 Ruijin Hospital, State Key Laboratory of Medical Genomics, Molecular Medical Center, Shanghai Institute of Endocrinology, Shanghai Jiao Tong University School of Medicine, Shanghai, China; University of Perugia, Italy

## Abstract

**Objective:**

The American Diabetes Association (ADA) recently published new clinical guidelines in which hemoglobin A1c (HbA1c) was recommended as a diagnostic test for diabetes. The present study was to investigate the association between HbA1c and cardiovascular risk, and compare the associations with fasting glucose and 2-hour oral glucose tolerance test (2 h OGTT).

**Research Design and Methods:**

The study samples are from a community-based health examination survey in central China. Carotid-to-femoral pulse wave velocity (cfPWV) and HbA1c were measured in 5,098 men and women.

**Results:**

After adjustment for age, sex, and BMI, the levels of HbA1c were significantly associated with an increasing trend of cfPWV in a dose-dependent fashion (P for trend <0.0001). The associations remained significant after further adjustment for blood pressure, heart rate, and lipids (P = 0.004), and the difference in cfPWV between the highest and the lowest quintiles of HbA1c was 0.31 m/s. Fasting glucose and 2 h OGTT were not associated with cfPWV in the multivariate analyses. HbA1c showed additive effects with fasting glucose or 2 h OGTT on cfPWV. In addition, age and blood pressure significantly modified the associations between HbA1c and cfPWV (P for interactions <0.0001 for age; and  = 0.019 for blood pressure). The associations were stronger in subjects who were older (≥60 y; P for trend = 0.004) and had higher blood pressure (≥120 [systolic blood pressure]/80 mmHg [diastolic blood pressure]; P for trend = 0.028) than those who were younger and had lower blood pressure (P for trend >0.05).

**Conclusions:**

HbA1c was related to high cfPWV, independent of conventional cardiovascular risk factors. Senior age and high blood pressure might amplify the adverse effects of HbA1c on cardiovascular risk.

## Introduction

Recently, the American Diabetes Association (ADA) published new clinical guidelines in which hemoglobin A1c (HbA1c) level, in addition to fasting glucose and 2-hour oral glucose tolerance test (2 h OGTT), was recommended as a diagnostic test for diabetes [1]. HbA1c is a marker of long-term glycemic exposure, reflecting an average blood glucose level over 2–3 month period of time. The cut-points for the diagnosis of diabetes are based on the presence of long-term complications. However, the current evidence for the ADA recommendation is largely from the studies on the relations between HbA1c and microvascular diseases especially retinopathy [2]. Even though macrovascular disease complications are more prevalent and the primary causes for mortality in diabetes, the data about the associations between HbA1c and macrovascular risk are surprisingly lacking.

Arterial stiffness is an established marker for early-stage atherosclerosis. Among various approaches to assess early-stage arterial stiffness, carotid-to-femoral pulse wave velocity (cfPWV) has been widely recognized a gold standard method [3], and independently associated with cardiovascular outcomes such as myocardial infarction, heart failure, and mortality [4]–[7]. Some recent studies associated HbA1c with arterial stiffness measured by cfPWV in patients with type 2 diabetes [8] and hemodialysis [9]. Studies examining the relation between HbA1c and arterial stiffness in the general population are sparse. In addition, fasting and post-challenge glucose concentrations were also related to accelerated stiffening of the elastic arteries that contributes to the excessive cardiovascular risk [7], [10], [11]. The effects of various glucose exposures are not perfectly concordant and may be independent [12]. However, little is known about the relative influence of HbA1c and fasting and post-challenge glucose on cardiovascular risk.

The aim of this study was to examine the associations between HbA1c as the marker of long-term glucose exposure and cfPWV in a large sample of Chinese adults, and to compare with the associations of fasting glucose and 2 h OGTT. We also assessed the modification effects of age, sex, obesity and blood pressure on the relations between glucose exposures and aortic arterial stiffness.

## Materials and Methods

### Study population

In the Cardiometabolic Risk in Chinese (CRC) Study, we performed a community-based health examination survey for 6,431 individuals (18–93 y) who were randomly selected from residents living in the urban area of Xuzhou, China, in 2009. Written consents were obtained from all the participants. The study was reviewed and approved by the ethics committee of the Central Hospital of Xuzhou, China. Among the participants, 5,514 individuals were measured for both cfPWV and HbA1c. For the present study, we excluded subjects with history of diabetes, or fasting glucose≥7.0 mmol/L, and/or 2 h OGTT ≥11.1 mmol/L, and/or HbA1c≥6.5% [12], [13]. In total 5,098 men and women were included in the final analyses. There was not significant difference in age and anthropometrics between individuals who were included and those who were not included in the analyses.

### Assessment of Carotid-to-femoral PWV

Carotid-to-femoral PWV in subjects at rest was measured using Complior device (Artech-Medical, Pantin, France) that allows pulse wave recording and automatic calculation of cfPWV with 2 transducers. The operator recorded in succession the right carotid and femoral waveforms. cfPWV is calculated as the distance between the two recording sites divided by the time delay between the feet of the two waveforms at each site. Sixteen cfPWVs were measured for each participant. After removing 6 extreme values (3 maximum and 3 minimum), an average value of cfPWV was calculated.

### Assessment of biomarkers and covariates

Venous blood sample was drawn from all subjects after an overnight fast (10 h). The blood was transferred into glass tubes and allowed to clot at room temperature. Immediately following clotting serum was separated by centrifugation for 15 min at 3,000 rpm. HbA1c was measured using high performance liquid chromatography (HPLC; HLC-723G7 hemoglobin HPLC analyzer, Tosoh Corp.) according to the standardized method. Participants with no history of diabetes underwent a 75-g oral glucose tolerance test (OGTT). Blood samples were drawn at 120 minutes after the glucose or carbohydrate load. Plasma glucose was measured using the hexokinase glucose-6-phosphate dehydrogenase method (Type 7600; Hitachi Ltd., Tokyo, Japan). The levels of total cholesterol, triglyceride, high-density lipoprotein cholesterol (HDL-C), and low-density lipoprotein cholesterol (LDL-C) were determined enzymatically using an autoanalyzer (Type 7600; Hitachi Ltd., Tokyo, Japan).

Height was measured to the nearest 0.5 cm without shoes and body weight was measured to the nearest 100 grams without shoes. Body mass index (BMI) was calculated as weight (in kilograms) divided by height (in meters) squared. Waist circumference was measured at the mid-point between the lowest rib margin and the iliac crest. Blood pressure was measured at the same time of cfPWV measurement after the subject had rested for at least 5 minutes with a mercury manometer by doctors. The subject's arm was placed at the heart level. Three measurements, 60 seconds apart, were taken. Systolic blood pressure (SBP) was defined as the average of the three SBP readings. Diastolic blood pressure (DBP) was defined as the average of the three DBP readings. The mean arterial pressure (MAP) was calculated as 2/3(DBP) +1/3(SBP).

### Statistical analyses

A linear regression model was used to evaluate associations between glucose exposures (HbA1c, fasting glucose, and 2 h OGTT) and cfPWV, adjusting for covariates. Glucose exposures were analyzed in quintiles. cfPWV was analyzed as the dependent variable, and levels of HbA1c, fasting glucose, or 2 h OGTT were analyzed as the independent variables. Tests for linear trend were calculated by assigning median value for each quintile of intake and treated as continuous variables. We adjusted for the potential confounding variables: age, sex, BMI, MAP, heart rate and lipids (total cholesterol, triglyceride, HDL-C and LDL-C). The effect modifications of age (<40, 40–59, ≥60 y), BMI (<25, ≥25 kg/m^2^), sex, and blood pressure (low vs high by the cutoffs SBP≥120 mmHg and DBP≥80 mmHg) were tested by introduction of the cross-products of the tested variables and HbA1c into the models. We used the SAS statistical package for all analyses (Version 9.1, SAS Institute, Cary, NC). All *P*-values are two-sided.

## Results

In total 5,098 men and women were included in the analyses. **Table** **1** presents the characteristics of the participants by the quintiles of HbA1c. Individuals with higher HbA1c had higher SBP, DBP, BMI, waist circumference, fasting glucose, 2 h OGTT, total cholesterol, triglyceride, and LDL-C, but lower HDL-C than those who were with lower HbA1c.

**Table 1:** Clinical characteristics of the participants by HbA1c levels.

	HbA1c in quintiles					P value
Variables	Q1	Q2	Q3	Q4	Q5	
	<5.06	5.06–5.15	5.16–5.33	5.34–5.52	5.53–6.49	
N of participants	1,181	1,043	1,068	772	1,034	
Age, years	43±10	46±12	49±12	52±13	56±13	<0.0001
Systolic blood pressure, mmHg	122±16	123±16	125±16	128±16	133±18	<0.0001
Diastolic blood pressure, mmHg	78±11	78±11	79±11	81±11	82±12	<0.0001
Heart rate, bpm	71±9	70±11	70±10	70±10	70±11	0.35
Waist circumference, cm	82.6±9.7	83.7±9.9	86.1±9.4	87.6±9.4	90.7±9.1	<0.0001
Body mass index, kg/m^2^	23.6±3.0	24.0±3.1	24.6±2.9	25.2±3.0	26.2±3.2	<0.0001
≥25 kg/m^2^	29.7%	34%	41.5%	52.6%	60.3%	<0.0001
Men, %	55%	56.1%	59.7%	56.6%	54.3%	0.1
Biochemical measures						
Glucose, mmol/L	4.79±0.41	4.88±0.38	4.99±0.45	5.07±0.44	5.43±0.62	<0.0001
2h OGTT, mmol/L	5.57±1.19	5.76±1.22	6.00±1.26	6.42±1.39	7.31±1.71	<0.0001
Total cholesterol, mmol/L	4.75±0.83	4.89±0.81	5.11±0.90	5.17±0.96	5.33±0.94	<0.0001
Triglyceride, mmol/L	1.33±1.22	1.37±1.03	1.59±1.58	1.69±1.50	1.88±1.78	<0.0001
HDL-C, mmol/L	1.29±0.31	1.26±0.30	1.25±0.30	1.21±0.29	1.19±0.28	<0.0001
LDL-C, mmol/L	2.74±0.7	2.94±0.69	3.08±0.81	3.16±0.75	3.26±0.81	<0.0001

Abbreviations: HDL-C, high density lipoprotein cholesterol; LDL-C, low density lipoprotein cholesterol;

OGTT, oral glucose tolerance test; bpm, beats per minute.

Data are mean ± standard deviations for the continuous variables and percentage for the categorical variables.

Linear regression model was used to test trend for continuous variables; χ^2^ test was used for the categorical variables.

We first examined the associations between three measures of glucose exposure (HbA1c, fasting glucose and 2 h OGTT) and cfPWV (**Table** **2**). In the models adjusting for age, sex and BMI, all the three measures were significantly associated with increasing trend of cfPWV (P<0.0001). When MAP was further adjusted, the associations for fasting glucose (P = 0.01), 2 h OGTT (P = 0.04), and HbA1c (P = 0.005) remained significant. When other covariates including heart rate, total cholesterol, TG, HDL-C and LDL-C were further adjusted, the associations for fasting glucose and 2 h OGTT were attenuated to be not significant, while the association for HbA1c remained significant (P = 0.004). In the fully-adjusted model, the difference in cfPWV between the highest and the lowest quintiles of HbA1c was 0.31 m/s.

**Table 2:** Associations of fasting glucose, 2h OGTT, and HbA1c with cfPWV (m/s).

	Glucose exposures in quintiles					P for trend
Models	Q1	Q2	Q3	Q4	Q5	
Fasting glucose						
Age, sex, and BMI adjusted	10.49 (0.05)	10.65 (0.05)	10.57 (0.05)	10.69 (0.05)	11.02 (0.05)	<0.0001
Further adjusted for blood pressure	10.62 (0.05)	10.71 (0.05)	10.58 (0.05)	10.64 (0.05)	10.86 (0.05)	0.01
Further adjusted for other covariates*	10.57 (0.06)	10.56 (0.06)	10.45 (0.06)	10.48 (0.06)	10.69 (0.06)	0.44
2h OGTT						
Age, sex, and BMI adjusted	10.62 (0.06)	10.57 (0.06)	10.58 (0.06)	10.68 (0.06)	11.04 (0.06)	<0.0001
Further adjusted for blood pressure	10.70 (0.05)	10.60 (0.06)	10.64 (0.05)	10.66 (0.05)	10.87 (0.06)	0.04
Further adjusted for other covariates*	10.56 (0.06)	10.56 (0.06)	10.44 (0.06)	10.52 (0.07)	10.69 (0.08)	0.56
HbA1c						
Age, sex, and BMI adjusted	10.59 (0.05)	10.63 (0.05)	10.56 (0.05)	10.69 (0.06)	10.95 (0.05)	<0.0001
Further adjusted for blood pressure	10.60 (0.05)	10.68 (0.05)	10.60 (0.05)	10.67 (0.06)	10.84 (0.05)	0.005
Further adjusted for other covariates*	10.46 (0.05)	10.55 (0.06)	10.47 (0.06)	10.61 (0.07)	10.77 (0.07)	0.004

* : Other covariates include heart rate, total cholesterol, triglyceride, HDL-C, and LDL-C.

cfPWV is presented as mean (standard error).

**Figure 1:**
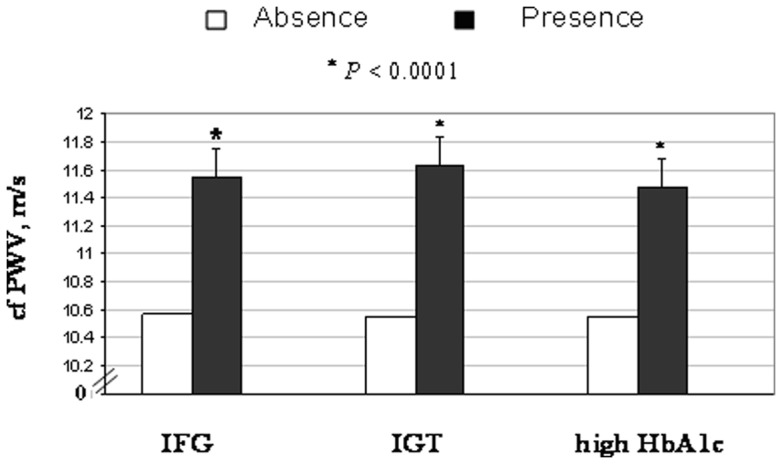
Geometric means of carotid-to-femoral pulse wave velocity (cfPWV, in m/s) by the presence of prediabetes status defined by IFG, IGT, and high HbA1c (5.7–6.4%). The analyses were adjusted for age, sex, BMI, MAP, heart rate and lipids (total cholesterol, triglyceride, HDL-C and LDL-C). Symbol ‘*’ represents significant difference (p<0.05) between the ‘absence’ and ‘presence’ groups of each marker.

According to the ADA recommendation [12], we defined ‘prediabetes’ by the cutoffs of the three glucose exposures, i.e. impaired fasting glucose (IFG, fasting glucose 5.6–6.9 mmol/L), impaired glucose tolerance (IGT, 2 h OGTT 7.8–11.0 mmol/L), and high HbA1c (5.7–6.4%) to represent an increased risk of diabetes but without fulfilling the criteria of diagnosis. The prevalence of IFG, IGT, and high HbA1c in this non-diabetic population were 12%, 14.3%, and 14.8%; respectively. The differences in cfPWV between individuals with IFG, IGT and high HbA1c and those without these abnormalities were 0.97, 1.08 and 0.92 m/s (P<0.0001; **Figure** **1**).

**Figure 2:**
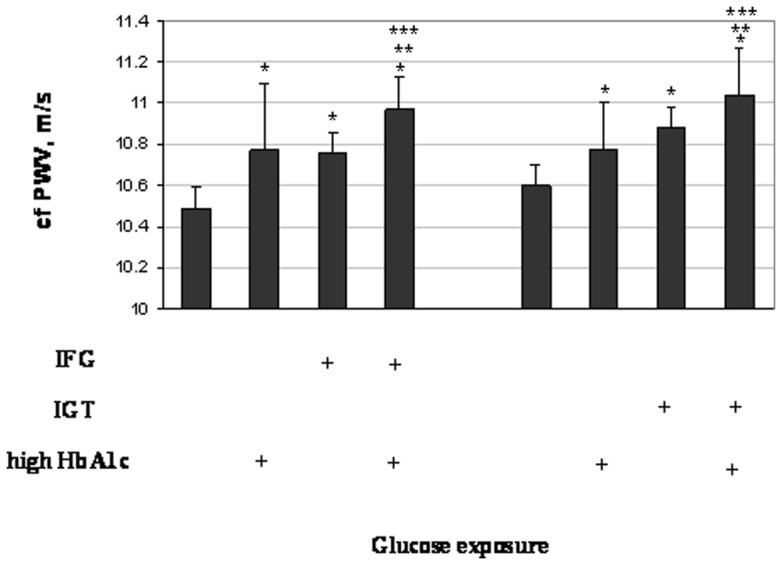
Geometric means of cfPWV (in m/s) by the combinations of high HbA1c (5.7–6.4%) with IFG and IGT. Symbol ‘+’ represents the presence of the corresponding glucose exposures. The analyses were adjusted for age, sex, BMI, MAP, heart rate and lipids (total cholesterol, triglyceride, HDL-C and LDL-C). ‘*’ represents significant difference (p<0.05) comparing individuals with high HbA1c and IFG/IGT with the normal subjects (without any of these abnormalities); ‘**’ represents significant difference (p<0.05) comparing individuals with high HbA1c and IFG/IGT with those who had only high HbA1c; and ‘***’ represents significant difference (p<0.05) comparing individuals with high HbA1c and IFG/IGT with those who had only IFG or IGT.

We then examined the additive effects of HbA1c with fasting glucose and 2 h OGTT on cfPWV (**Figure** **2**). In the analyses adjusting for age, sex, and BMI, as compared with individuals with fasting glucose <5.6 mmol/L and HbA1c <5.7%, those with high HbA1c only, with IFG only, and with both IFG and high HbA1c had 0.28 (P = 0.03), 0.27 (P = 0.015) and 0.48 m/s (P = 0.0003) higher cfPWV. Similarly, as compared individuals with 2 h OGTT <7.8 mmol/L and HbA1c <5.7%, those with high HbA1c only, with IGT only, and with both IGT and high HbA1c had 0.18 (P = 0.10), 0.28 (P = 0.008) and 0.44 m/s (P = 0.0001) higher cfPWV. Further adjustment for other covariates did not materially change the results. Our data also indicate that individuals of both high HbA1c and IFG or IGT had significantly higher levels of cfPWV compared with those who only had high HbA1c (p = 0.036 and 0.03; respectively; or those only had IFG (p = 0.02); or those only had IGT (p = 0.04).

**Figure 3:**
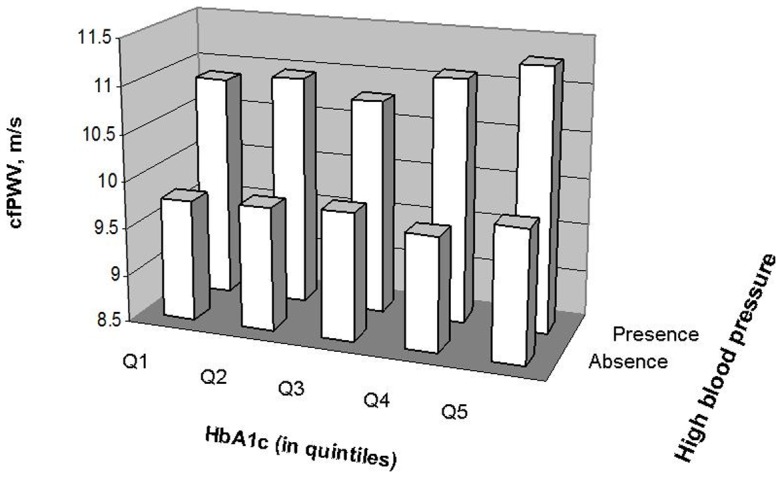
The geometric means of cfPWV (in m/s) by according to HbA1c (in quintiles) by the presence/absence of high blood pressure defined by SBP ≥120 **mmHg and DBP ≥80** **mmHg.** The analyses were adjusted for age, sex, BMI, heart rate and lipids.

Pulse wave velocity is strongly associated with age and blood pressure, and is also related to sex and BMI [14]–[16]. Therefore, we examined whether these variables modified the relation between HbA1c and cfPWV. We found significant interactions between HbA1c and age (<40, 40–59, ≥60 y; P<0.0001) and blood pressure (low vs high by the cutoffs SBP≥120 mmHg and/or DBP≥80 mmHg [17]; P = 0.019). The associations between HbA1c were stronger in participants with age ≥60 y (P for trend = 0.004) than in those who were younger (P for trend >0.05) (**Table** **3**); and were stronger in participants with high MAP (P for trend = 0.028) than those with low MAP (P for trend >0.05) (**Figure** **3**). Although the associations between HbA1c and cfPWV appeared to be more significant in women and in subjects with BMI <25 kg/m^2^ than in men and in those with BMI ≥25 kg/m^2^, the tests for interactions with sex and BMI were not significant (**Table** **3**).

**Table 3:** Stratified associations between HbA1c and cfPWV (m/s) by sex, age and BMI.

	HbA1c in quintiles					P for trend	P for interaction
	Q1	Q2	Q3	Q4	Q5		
Age							
<40 y; N = 1143	10.03 (0.07)	10.05 (0.09)	9.93 (0.11)	10.07 (0.14)	10.26 (0.18)	0.71	<0.0001
40 to 59 y; N = 2932	10.53 (0.07)	10.60 (0.07)	10.39 (0.07)	10.47 (0.09)	10.51 (0.09)	0.59	
≥60 y; N = 1023	12.40 (0.55)	12.00 (0.55)	12.91 (0.39)	12.86 (0.40)	13.50 (0.27)	0.004	
Sex							
Men, N = 2871	10.77 (0.07)	10.90 (0.07)	10.72 (0.07)	10.86 (0.09)	11.10 (0.09)	0.06	0.88
Women, N = 2227	9.85 (0.07)	9.85 (0.08)	9.98 (0.10)	10.13 (0.13)	10.05 (0.13)	0.03	
BMI							
<25 kg/m^2^; N = 2918	10.26 (0.06)	10.42 (0.07)	10.33 (0.08)	10.39 (0.11)	10.62 (0.12)	0.025	0.42
≥25 kg/m^2^; N = 2180	10.82 (0.09)	10.76 (0.10)	10.66 (0.09)	10.88 (0.10)	10.92 (0.09)	0.32	

Analyses were adjusted for age, sex, BMI, total cholesterol, triglyceride, HDL-C, LDL-C, blood pressure, and heart rate but not the strata variable. cfPWV is presented as mean (standard error).

## Discussion

In this study of a large sample of Chinese adults, long-term glycemic exposure, measured by HbA1c, was significantly associated with higher cfPWV, independent of other cardiovascular risk factors. We found that fasting glucose and 2 h OGTT showed additive effects with HbA1c on cfPWV. In addition, age and blood pressure significantly modified the associations between HbA1c and cfPWV.

Carotid-femoral pulse wave velocity, a gold-standard measure of intrinsic stiffness of the aortic wall, is an important predictor of cardiovascular disease risk [4], [18], [19]. Our findings are consistent with some previous studies. Matsumae et al. reported that HbA1c level was an independent determinant of cfPWV in hemodialysis patients with and without diabetes [9]. The association between HbA1c levels and increased arterial stiffness (measured by brachial-ankle pulse wave velocity [baPWV]) was recently observed in patients with type 2 diabetes [8]. Our data indicate that the long-term glucose exposure HbA1c may lead to increased arterial stiffness in non-diabetic individuals. The precise mechanisms how chronic glucose exposure may affect arterial stiffening are not fully understood. It was documented that BMI, blood pressure, heart rate, total cholesterol, LDL-C, HDL-C and triglyceride were related to cfPWV [15], [16], [20], [21]. In our study, adjustment for these potential risk factors did not change the associations of HbA1c with cfPWV, suggesting that the effects of HbA1c are not mediated by these metabolic changes and likely to be through other independent pathways.

Individuals with prediabetes defined by the three measures of glucose exposure HbA1c, fasting glucose and 2 h OGTT all had significantly higher cfPWV. Our data indicate that, however, the changes in arterial stiffness were better associated with long-term glycemic exposure HbA1c than single measures of fasting glucose and 2 h OGTT on the continuous scales. Though highly correlated, these various measures for glucose exposure may convey different information regarding their relations with cardiovascular risk. Our data are consistent with previous studies in which HbA1c was a better predictor of cardiovascular disease than fasting glucose [22] and post-challenge glucose levels [23]. In addition, HbA1c has shown more consistent associations with retinopathy than fasting glucose level [2]. HbA1c value is a more stable biological index for long-term glycemia exposure than fasting glucose, which fluctuate within and between days and is therefore not a clear indicator of general glycemia.

Various mechanisms have been proposed to link glucose exposure with development of atherosclerosis. Long-term high levels of circulating glucose lead to formation of advanced glycation endproducts (AGE), which result from non-enzymatic protein glycation forming irreversible cross-links in stable tissue proteins [24]. Matrix in the blood vessel wall is steadily reduced from exposure to AGE [25]. In addition, high AGE is known to impact endothelial function by quenching NO, enhancing the generation of reactive oxygen species (ROS), and induction of inflammation. All these alterations may contribute to development and progression of atherosclerosis [24], [26]. It has been acknowledged that various glucose exposures are not perfectly concordant regarding their relations with disease outcomes [12]. Our study suggests that different measures of glucose exposure might have additive effects on arterial stiffness. Although these measures have been individually related to cardiovascular risk [8], [10], the mechanisms underlying their joint effects are not unequivocally clarified. These various measures may reflect distinct abnormalities in glucose metabolism. High fasting glucose detects fasting hyperglycaemia, which is more typical of pancreatic β cell dysfunction; whereas high 2 h OGTT reveals post-prandial hyperglycaemia that is more closely associated with insulin resistance [27].http://pmj.bmj.com.ezp-prod1.hul.harvard.edu/content/86/1021/656.long – ref-11 HbA1c provides a weighted average of blood glucose for the lifespan of an erythrocyte and the measure may reflect recent changes in diet or treatment [28]. We postulate that at least parts of the pathways linking these different markers to cardiovascular conditions are not overlapped. Therefore, when different markers are considered jointly, their effects would appear additive. Our findings of the additive effects of different measures of glucose exposures highlight the importance to measure long-term glucose exposure in addition to fasting glucose and 2 h OGTT in characterizing individuals at high risk of diabetes and cardiovascular complications.

Arterial stiffness is strongly associated with age and blood pressure [21]. The data from the present study showed that these two factors significantly modified the associations between HbA1c and cfPWV, and stronger associations were observed in the individuals who were older and had higher blood pressure. A synergistic effect between raised blood pressure and raise plasma glucose on arterial stiffening was reported in middle-aged Japanese men [29]. Though the mechanisms underlying such an additive effect remain not clear, some studies have shown that the coexistence of hyperglycemia and high blood pressure might augment the production of advanced glycation end products and deteriorate endothelial dysfunction [29], [30]. Future studies are needed to investigate the mechanisms underlying the additive effects between HbA1c and blood pressure and aging.

The sample size of this study is large, which ensures sufficient power to detect the moderate effects of glucose exposures on arterial stiffness and interactions between HbA1c and other risk factors. Our study is cross-sectional in design. Therefore, a causal relation between HbA1c and arterial stiffness could not be derived. In addition, the study was performed in a Chinese population. Further studies in other populations of different ethnicities are warranted to verify our findings. Moreover, we used the standardized method in measurement of HbA1c. However, it has been argued that the use of HbA1c for diagnosing diabetes has some limitations [31]. For example, the measurement of HbA1c level might be influenced by various medical conditions, such as kidney failure, chronic excessive alcohol intake, acute or chronic blood loss and liver failure. Although we have carefully excluded patients with chronic diseases from the analysis, it is still possible some conditions that were not assessed in our study might influence the associations.

In summary, in the present study of non-diabetic Chinese adults, the marker of long-term glucose exposure HbA1c showed stronger association with aortic arterial stiffness than fasting glucose and 2 h OGTT. Various measures for glucose exposures might additively affect arterial stiffness. In addition, we found that senior age and high blood pressure might amplify the effects of chronic glucose exposure on the cardiovascular risk.
